# Spontaneous attention-capture by auditory distractors as predictor of distractibility: a study of domestic horses (*Equus caballus*)

**DOI:** 10.1038/s41598-017-15654-5

**Published:** 2017-11-10

**Authors:** C. Rochais, S. Henry, M. Hausberger

**Affiliations:** 1 Université de Rennes 1, UMR CNRS 6552 - Laboratoire Ethologie Animale et Humaine-EthoS- Station Biologique, 35380 Paimpont, France; 2 CNRS- UMR 6552 Université de Rennes 1, - Laboratoire Ethologie Animale et Humaine- 263 avenue du général Leclerc, 35042 Rennes cedex, France

## Abstract

Distractibility (*i.e*. individual distraction from his ongoing activity) is thoughts to affect daily life. The present study develops an easy way to assess inter-individual variations of distractibility of an animal model, the domestic horse. We developed the ‘distractibility test’ (DT), based on auditory stimuli, a major source of distraction in daily life. We hypothesized that the broadcast of unusual sounds would provide a reliable source of distraction and that the responses to these unusual sounds would yield a good estimation of a horse’s level of distractibility. Validity of the DT was assessed by comparing the subjects’ interest towards the sound in this test to their attentional state in experimental visual attention tasks and in a working task. Our results showed inter-individual differences in response to the stimuli, with consistency over time. The subjects’ responses to this DT were negatively correlated to their attentional skills in separate experimental tests and in a working task. This is to our knowledge the first ‘real-world’ estimate of an animal’s distractibility in its home environment that could potentially be adapted for humans.

## Introduction

The ability to attend to one source of information while ignoring or excluding other aspects of their environment (*i.e*. attention)^1,2^ is crucial for individuals during their daily activities. Efficiency in a task requires attention to be focused exclusively on information relevant to the task^3^. However, some individuals can be distracted from their ongoing activity by irrelevant external stimuli (which refers to one of the definitions of distractibility)^4^. In humans, distractibility can have deleterious consequences, causing for instance work accidents^5^ while in animals it may lead to less appropriate decision taking in contexts such as predator detection^6^, social cooperation, competition, communication or social learning (*e.g*.^7,8^). Watching for predators and monitoring the activities of nearby conspecifics are often important for survival and reproductive success^6^.

Understanding the reasons of inter-individual variations of distractibility is therefore an important issue. However, this question is still highly debated and unsolved. To date, in humans most studies used questionnaires and/or response-competition paradigms (*i.e*. distraction by extraneous external stimuli presented during a task performance) to assess distractibility^5^. Indeed, most experimental reports focused on experiments presenting visual or auditory distractor stimuli during a task. In such context, Forster & Lavie^3^ (2014) showed that the estimation of distractibility during a task (*e.g*. a response-competition paradigm where participants made speeded forced-choice responses to one of two target letters (X or N) while attempting to ignore a distractor letter) was more reliable when irrelevant distractors to the task (*e.g*. a distractor image of Superman) were used rather than relevant (to the task) distractors (*e.g*. distractor letters). The authors argue that measuring distraction by salient yet task-irrelevant external stimuli is more appropriate to parallel a form of distraction that, like distraction from task-unrelated thoughts, is common in daily life. On the other hand, most common distractors in daily life are auditory. Contrary to visual stimuli, sound stimuli can be perceived whatever an individual’s activity. However, intriguingly, most researches focused on visual distraction^5^. Self-evaluations of distractibility are often performed using the “cognitive failure questionnaire” creating an index of everyday distractibility (the higher the score, the more prone to these cognitive slips the person reports to be)^9^. The results obtained through this questionnaire show correlations with occurrence of lost computer work, traffic accidents and performances in an auditory attention task (without distractors) in the laboratory^5^, suggesting individual distractibility consistency between situations (*i.e*. questionnaire, daily life and laboratory task).

As distractibility seems to present some intra-individual consistency, direct observations of distraction in daily life (*i.e*. the ‘real world’) should be a promising way to avoid potential biases due to self-evaluation or experimental settings. This approach could also open the way for comparative research on distractibility by enabling comparisons among different animal models, which are still very scarce (*i.e*. most studies are done in rodents^10^).

Predicting distractibility is nevertheless important for both humans (to promote safety measures or attentional education) and domestic working animals such as working dogs or horses (to also promote attentional education and better work performances) *e*.*g*.^11^. For working horses, distractibility is a major problem both in terms of performances and of security for handlers and riders. Less attentive horses are known to learn less well^12,13^ as do horses distracted by human presence^14^ or in an altered welfare state^15^.

This study performed in horses (a species known for reacting easily to sounds *e.g*.^16^ and whose attentional state and direction of attention can be easily identified through postural cues^12,17,18^) proposes a short simple test based on the broadcast of novel sounds that could be used to assess distractibility in the daily life environment. We hypothesized that unexpected unusual sounds would provide a reliable source of distraction and that individuals’ responses (*e.g*. reaction time and duration of attention) to these unusual sounds would yield an estimation of their distractibility. The validity of the distractibility test (DT) was assessed by comparing the subjects’ attention capture by the auditory distractors in this test to their attention abilities scored previously in two experimental visual attention tasks (see below), as well as during a lunging training (work) task. The two visual attention tests were: *i)* the visual attention test (VAT) that evaluates the reactions of a horse facing the display of a novel moving visual stimulus (*i.e*. the light of a laser pointer)^19^, and *ii)* the ‘five-choice serial reaction time task’ (5-CSRTT), a validated test of attention in rodents^20,21^ adapted for horses^19^.

## Results

Twelve adult mares (5 to 17 years old, 8.9 ± 1.1) living under the same conditions and from Anglo-Arabian (n = 10) and French Saddlebred (n = 2) breeds were tested in their usual individual home stalls. Each of the two novel unfamiliar sounds (one element of a whale song, one whinny of an unknown horse) was broadcast for 3 seconds once a day with an inter-stimuli interval of at least 5 minutes for four consecutive days (D1 to D4) while mares were performing their usual activities (*i.e*. feeding, resting). Video recordings of their behaviours were analysed subsequently to record the reaction time (*i.e*. time between auditory stimulus broadcast and the horse’s first change in behaviour) and the duration of interest (*i.e*. duration of its first fixed gaze) towards the stimuli. The measures did not vary significantly between the two sound stimuli whatever the test day (Wilcoxon signed-rank tests: P > 0.05 in all cases for reaction time and duration of attention) and responses to both stimuli were correlated (Spearman’s correlation test, N = 12, *e.g. duration of attention towards the sounds D1:* rs = 0.71, P = 0.009) indicating that the subjects responded similarly to both stimuli. Therefore, the data for both stimuli were pooled for further analysis. No fear reactions (alert posture, flight…) were observed for any of the stimuli.

The subjects had previously been submitted to two experimental visual attention tests: the visual attention test (VAT) and the ‘five choice serial reaction time task’ (5-CSRTT), and to a work task. The VAT was performed on two consecutive days and gazes towards the visual stimulus were estimated by reaction time, duration and fragmentation (ratio number of gaze sequences/total duration of gazes, the higher the ratio is, the more fragmented attention is) of attention^19^. In the 5-CSRTT^21^ the test subject had to identify correctly which of the five openings has been briefly illuminated and then approach it to get access to a food reward. The 5-CSRTT was performed on two consecutive tests days. The attentional capacities of the subject were assessed by the ‘accuracy’ (*i.e*. correct responses/total responses) of choosing the correct (*i.e*. illuminated) opening and reaction time. The work task was lunging (*i.e*. horses had to obey an experimenter’s vocal command); at the end of the training (day 10) inattentive behaviours (*i.e*. sudden unrequested gait changes) and reaction times were recorded^22^.

### Distractibility test (DT)

The number of horses reacting (*i.e*. by a behavioural change) decreased gradually over test days from all (12) mares on day 1 to only 4 (out of 12) on days 3 and 4 (Chi-square test, df = 1, *D1/D2*:*X* ^2^ = 8.2, P = 0.004; *D1/D3*:*X* ^2^ = 12.6, P = 0.0004; *D1/D4*:*X* ^2^ = 14.3, P = 0.001). This indicated habituation and thus only data from D1 and D2 will be considered further. Only 4 horses remained interested towards the stimuli over time. Interestingly, they were the youngest animals (*i.e*. less than 10 years old) but no correlation with age was found (Spearman’s correlation test, N = 12, P > 0.05 for all).

On the first day, reaction times were between 0.2 and 15.6 seconds (median value = 2.2 s), but inter-individual variations were nevertheless important. We used the ‘coefficient of variation’ (CV = standard-deviation/mean), which has been shown to quantify the level of variation and thus compare this level between variables, to estimate the degree of individual variations on the different variables^23^. This coefficient was of 161% for reaction times but of 62% for the duration of the first attention sequence towards the sounds (range: 0.9 to 12.8 s; median value = 8.4 s), showing a higher inter-individual variation for reaction times.

### Relationships between interest towards the sounds during the distractibility test (DT) and the attention level during the attention tests (VAT and 5-CSRTT)

The VAT test had revealed clear individual differences especially in the reaction time which varied from 0.1 to 300 seconds, the number of attention sequences shown during the 5- min test (from 0.0 to 17.0) and the pattern of attention with an attention fragmentation index that ranged from 0.0 to 0.5. Differences appeared however between the first day of testing on one hand (suggesting that we might have measured different attentional characteristics on D1 due to the novelty of the stimulus) and the following test on day 2 on the other hand: attention structure on the second day was predictive of learning abilities, attention performances in the 5-CSRTT and at work^19^.

**Figure 1:**
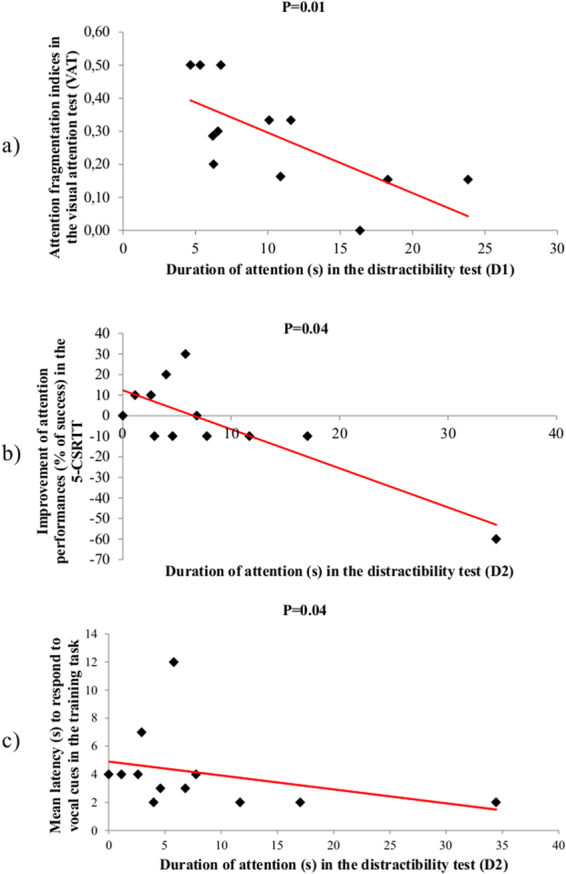
Correlation between distractibility test (DT) data (durations of attention or reaction times) and (**a)** attention fragmentation (*i.e*. number of attention sequences/total duration of attention) in the visual attention test (VAT); (**b)** improvement of attention performance between Test 1 and Test 2 in the 5-CSRTT (*i.e*. the higher the score, the more the horse was attentive in Test 2 than in Test 1); (**c)** attention in the training (lunge working) task measured via mean latency to respond to vocal cues. Longer duration of attention in the DT predicted less attention in the VAT, the 5-CSRTT and the work task.

There were clear correlations between both tests, DT and VAT: the duration of interest towards the auditory stimuli on D1 was negatively correlated with VAT attention fragmentation indices on D2 (Spearman’s correlation test, N = 12, rs = −0.70, P = 0.01). The longer the subjects were interested by the auditory stimuli on D1, the less fragmented attention was in the VAT (Fig. 1a) (Table 1). Correlations were especially clear on D2: the longer the subjects were interested by the auditory stimuli on D2, the less reactive they were to the visual stimulus on D2 (Spearman’s correlation test, N = 12, rs = 0.72, P = 0.008).

**Table 1:** Attention variables.

	Variables in the VAT	Reaction time in the DT		Duration of attention in the DT	
		D1	D2	D1	D2
D1	Reaction time (s)	Rs = −0.11 P = 0.71	Rs = −0.29 P = 0.35	**Rs =** −**0.63** P** = 0.02**	Rs = 0.10 P = 0.73
	Total duration (s)	Rs = 0.10 P = 0.75	Rs = 0.26 P = 0.40	Rs = −0.04 P = 0.89	Rs = 0.10 P = 0.75
	Mean duration of sequences (s)	Rs = −0.12 P = 0.70	Rs = −0.11 P = 0.72	Rs = 0.04 P = 0.89	Rs = 0.25 P = 0.41
	Number of sequences (#)	Rs = 0.07 P = 0.82	Rs = 0.26 P = 0.41	Rs = −0.03 P = 0.92	Rs = 0.05 P = 0.85
	Fragmentation index	Rs = 0.20 P = 0.51	Rs = 0.03 P = 0.92	Rs = 0.33 P = 0.28	Rs = −0.21 P = 0.49
D2	Reaction time (s)	Rs = −0.53 P = 0.07	Rs = −0.25 P = 0.42	Rs = −0.08 P = 0.78	**Rs = 0.72** P** = 0.007**
	Total duration(s)	Rs = 0.50 P = 0.09	Rs = 0.35 P = 0.25	Rs = 0.42 P = 0.16	**Rs =** −**0.56** P** = 0.05**
	Mean duration of sequences (s)	Rs = 0.20 P = 0.52	Rs = 0.05 P = 0.86	Rs = 0.40 P = 0.18	Rs = 0.23 P = 0.46
	Number of sequences (#)	Rs = 0.51 P = 0.08	Rs = 0.44 P = 0.14	Rs = 0.24 P = 0.44	**Rs =** −**0.57** P** = 0.05**
	Fragmentation index	Rs = 0.26 P = 0.40	Rs = 0.41 P = 0.18	**Rs =** −**0.70** P** = 0.01**	Rs = 0.02 P = 0.94

Correlations between attention variables in the DT (D1, D2) and attention variables in the VAT (D1, D2). Significant correlations are in bold (Spearman correlation test).

Similar results were obtained with the second attention test: the subjects which showed more interest towards the auditory stimuli in DT performed also less well in the 5-CSRTT. The reaction time (*i.e*. the more time they required to react) to the auditory stimuli on the first day was positively correlated with an increased performance in the 5-CSRTT (difference of % of accuracy from Test 1 to Test 2; Table 2). Moreover, the longer (in duration) was its interest for the auditory stimuli during the DT at D2, the less performant (percentage of accuracy) it was, especially at D1 of the 5-CSRTT (Fig. 1b) (Table 2).

**Table 2:** Attention variables.

5-CSRTT	Reaction time in the DT		Duration of attention in the DT	
	D1	D2	D1	D2
Learning ability	Rs = −0.26 P = 0.41	Rs = −0.41 P = 0.17	Rs = −0.15 P = 0.62	Rs = 0.06 P = 0.84
Accuracy T1	Rs = −0.11 P = 0.72	Rs = 0.19 P = 0.53	Rs = −0.25 P = 0.41	**Rs =** −**0.59** P** = 0.04**
Accuracy T2	Rs = 0.43 P = 0.16	Rs = −0.01 P = 0.96	Rs = 0.01 P = 0.95	Rs = −0.07 P = 0.80
Improve perf T1-T2	**Rs = 0.56 P = 0.05**	Rs = −0.01 P = 0.95	Rs = 0.38 P = 0.21	**Rs =** −**0.58** P** = 0.04**

Correlations between attention variables in the DT (D1, D2) and attention variables in the 5-CSRTT. Significant correlations are in bold (Spearman correlation test).

### Relationships between interest towards auditory stimuli during the distractibility test (DT) and attention level during the training (work) task

The duration of interest towards auditory stimuli on D2 of the DT was negatively correlated with the latency to respond to vocal cues in the work task (Fig. 1c, Table 3). Therefore, horses more easily distracted during the DT were less attentive during the working task, despite the fact that the orders were given vocally.

**Table 3:** Attention variables.

Training task	Reaction time in the DT		Duration of attention in the DT	
	D1	D2	D1	D2
Gate change (#)	Rs = −0.22 P = 0.47	Rs = −0.46 P = 0.12	Rs = 0.22 P = 0.48	Rs = 0.17 P = 0.59
Latency to respond to vocal cues	Rs = −0.20 P = 0.52	Rs = −0.14 P = 0.64	Rs = 0.24 P = 0.43	**Rs =** −**0.57** P** = 0.04**

Correlations between attention variables in the DT (D1, D2) and attention variables in the working task. Significant correlations are in bold (Spearman correlation test).

## Discussion

The test developed here appears a simple and efficient way to estimate individual differences in horses’ distractibility. This approach is innovative as it is based on horses’ spontaneous responses to auditory stimuli in their home environment. Evaluating subjects’ distractibility in their daily life, *i.e*. in the ‘real world’, appears to be a useful predictive tool as the interest towards these auditory stimuli was negatively correlated to individual attentional capacities in other experimental (*i.e*. VAT; 5-CSRTT) and working situations (*i.e*. training lunge task). Interestingly, the fact that no difference was found between reactions to unfamiliar conspecific and whale auditory stimuli seems to show that inexpectancy and novelty of a stimulus ensured their distraction potency: it supports the suggestion that an irrelevant event is difficult to ignore, particularly when it is completely without meaning to the subject at hand, and hence, has higher potential distraction effect^3^.

Such distractibility test mimics a form of distraction caused by irrelevant and unusual sounds, events that are common in humans’ and animals’ daily life^3^. The method is simple because the DT is needed to be replicated only two following days to show subject distractibility tendency. DT could also open a way for comparative research on distractibility by enabling the investigation of model animals’ spontaneous changes in behaviour. Moreover, the use of auditory stimuli as distractors appears relevant as most distractors in daily life are auditory^5^ and sound stimuli can be perceived whatever an individual’s activity. Our results also promote the idea that more ethological studies in the home environment should lead to a better understanding of the processes involved in humans. Thus, this DT could be a promising tool, easy to use rapidly to predict both animals’ and humans’ distractibility.

Clear inter-individual differences of distractibility (*i.e*. some mares responded faster or paid more interest to the sounds than others) were identified. As in previous studies, these differences are partially explained by subjects’ age, as young horses were more prone to be distracted over days. The results of our study also reveal some intra-individual consistency of distractibility between situations (*i.e*. between daily life, experimental and working situations, whale *versus* horse vocalisations) and over successive days. As previously shown by human studies, this study confirms that subjects’ distractibility in daily life can predict distractibility in both experimental and working contexts^5^, while studies of individual consistency of animals’ distractibility are still scarce to date. Some authors, using questionnaires, showed consistency of dogs’ distractibility related to affection demands addressed to humans and aggression of other dogs^24^. Some horses seemed to be more distracted by human presence than others^14^.

Predicting distractibility is nevertheless important for both humans and domestic working animals. The ability to ignore irrelevant distracting stimuli is relevant to everyday life, as the effects of distraction can have a range of consequences, some that impair the quality of life^25^. Furthermore, attention is considered to underlie a variety of cognitive processes, such as learning and memory^26^. Proneness to distractibility, inattentiveness, or failure of attentional control^4^ may lead to poorer learning performances as shown by horses who performed less well in the 5-CSRTT or by horses with an altered welfare^15^ who learned less well in a training task^12^. Humans’ self-evaluations through the ‘cognitive failure questionnaire’ showed that distractibility was correlated with occurrences of lost computer work, of traffic accidents, and also lower performances in auditory attention tasks in the laboratory^5^.

This is to our knowledge the first estimate of spontaneous distractibility in an animal model tested in its home environment, *i.e*. in the real world situation. This may well be a promising approach to predict human distractibility.

## Material and Methods

### Ethical note

This study was approved by the French national ethics committee (Animal utilisation protocol number: 33, 12-2013-12). Experiments complied with the current French laws related to animal experimentation and were in accordance with the European directive 2013/118/CEE. This experiment included only behavioural observations and non-invasive contacts with the horses. Animal husbandry and care were under management of the staff of the research station in Chamberet, France.

### Subjects

This study, conducted at the ‘Station Expérimentale des Haras Nationaux’ (French National Studs – ‘Institut Français du Cheval et de l’Equitation’) in November 2013, included twelve 5 to 17 years old (8.9 ± 1.1), Anglo-Arabian (n = 10) and French Saddlebred (n = 2) breeding mares (10 out of 12 were in the early stages of gestation, *i.e*. ≤ 5 months). In order to test validity of the DT, all the subjects were of the same sex and from the same site to limit potential biases related to sex and living conditions. They had been born and raised under the same living conditions since birth: they were kept in individual stalls at parturition period (April-July), as one group in large pastures in summer (August-October) and in large communal stalls during winter (November-March). Human interventions were limited to routine procedures (occasional veterinary care, weighing once a week, pellets and hay distribution twice a day in winter and moving from one pasture to another once a week in summer).

### The distractibility test (DT)

#### Test setting

Horses were exposed to two auditory stimuli broadcasted at 07:30am for 3 seconds at 70db, each one once every day (order of broadcast counterbalanced according to test days), for 4 consecutive days. As our aim was to assess the importance of auditory stimuli as potential distractors, we chose unusual (in this context) auditory stimuli, *i.e*. a totally unknown heterospecific vocalisation (*i.e*. an element of a blue whale’s song) and an unfamiliar conspecific vocalisation (*i.e*. whinny of an unfamiliar horse as horses are known to react strongly to unknown whinnies^27,28^). These two stimuli differed clearly in terms of acoustic features (*i.e*. frequency).

Mares were tested in their home stalls (4 × 4 m). The loudspeaker (Logitech Pure-Fi Mobile®) was placed on a 70 cm high table (visible to promote the “irrelevant” aspect of the stimuli) in the middle of the corridor separating two rows of stalls (*i.e*. 3 m away from the individual stalls) (following Noble *et al*.’s^16^ procedure). Tests were videotaped using an ‘axis M1054-W camera®’ mounted on the top of each horse’s stall. Each camera was 3 meters height, on the middle of the stall wall towards the stimuli broadcast. The wide camera angle offers the opportunity to record horses’ reactions.

#### Quantitative Analyses

Predefined traits of attention to sound stimuli were extracted by one single experimenter (CR) from videos using continuous focal sampling^29^. The experimenter (CR) also conducted both visual attention tests and the work task, but she had not yet analysed the results of the other tests before analysing the DT which means that she was somewhat blind to the potential correlations.

**Figure 2:**
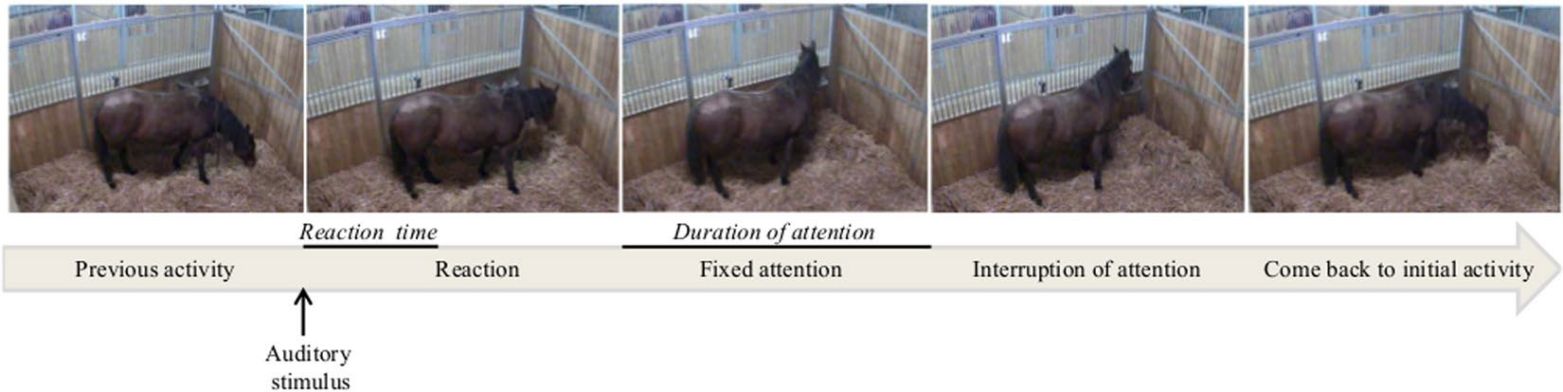
Detailed ethogram of horses’ attention towards the auditory stimuli: a reaction was defined as any change in the ongoing activity or any body movement of ears, head, neck or all body. Attention was characterized by the horses standing with their body completely immobile and their fixed visual and auditory sensory organs (*i.e*. use of their binocular visual field, pointing both ears towards the stimulus source) converging towards the stimulus. Interruption of attention towards the auditory distractors was indicated by the horse’s ears or head starting to move and the horses came back to their initial activity. This detailed behaviours allow the measurement of the reaction time *i.e*. time between the auditory stimulus broadcast and the horse’s first change in behaviour and the total duration of attention.

The reaction time, *i.e*. time between the auditory stimulus broadcast and the horse’s first change in behaviour, of each horse was measured. A reaction was defined as any change in behaviour (*e.g*. change in the ongoing activity or any body movement of ears, head, neck or all body)^27,28^ (Fig. 2). The subjects that did not react within the time limit (5 min) were given the maximum latency score of 300 seconds. This time limit was chosen on the basis of earlier studies^19,30^ and the finding that after 5 minutes, horses go back to their activities. We then measured for each horse the duration of its interest (*i.e*. duration of its first fixed gaze) towards the stimulus source which is easily identified: indeed, when horses ‘fixed’ their attention^27,31^: they stand with their body completely immobile and their visual and auditory sensory organs (*i.e*. use of their binocular visual field^17^, pointing both ears towards the stimulus source^17,18^) converging towards the stimulus^32^. Because the stimuli were not fear-inducing (neutral unknown sounds), even when the horse presented this fixed attention towards the sound source, it was never observed in an alert characteristic posture (‘vigilance’), that is with an elevated neck or even a raised tail^33^, which would be equivalent to the vigilance postures observed in groups of birds with predation risk^8^. Here the neck remained somewhat above horizontal and no flight, or mere avoidance behaviour was recorded. Therefore the horses were more interested than afraid of the sounds. Interruption of attention towards the auditory distractors was indicated by the horse’s ears or head starting to move^32^ and the horses came back to their initial activity (Fig. 2).

### The visual attention test

The horses were tested individually in their home stalls. Subjects could move freely. The green light of a laser pointer (Laser point, PEARL®) was projected on the subject’s stall door for 5 minutes, always by the same experimenter (CR) who stood motionless in the middle of the stall, facing the stall door, on the horses’ the left side and level with the horse’s shoulder. Horses were tested once a day on two consecutive days. Tests were videotaped using an axis M1054-W camera® mounted on the top of the stall wall and data were transcribed later.

Using continuous focal sampling^29^, we recorded subjects’ gazes (≥1 second^34^). Any movement (*e.g*. head) was considered as ending a gaze sequence. The structure of visual attention was estimated by the following scores:

- number of gaze sequences
- duration (in seconds) of each gaze sequence
- total duration (in seconds) of gazes at the stimulus (*i.e*. total of durations of all gazes during the 5-minute test)
- an index of attention fragmentation calculated by dividing the number of gaze sequences by total duration of gazes during the 5-minute test (number/seconds): the higher the index, the more attention was fragmented.

### The 5-choice serial reaction time task (5-CSRTT)

The 5-CSRTT was first adapted for rodents by Carli *et al*.^20^ from a test for human subjects^35^ and is to date regularly used to assess different aspects of rodents’ attention^21^. Slightly different versions of the task exist for monkeys^36^. The test subject has to identify correctly which of the five openings has been briefly illuminated and then approach it to get access to a food reward (*i.e*. associative learning, operant conditioning). We adapted the 5-CSRTT to horses where subjects have a choice between five chests containing food and an orange light was fixed on the front of the chests. The discriminative stimulus was a brief (0.5 s) illumination of one of the five orange lights. Horses learned to go towards the illuminated chest (correct response), to open the chest lid and obtain access to food (*i.e*. only one mouthful was allowed). When a horse made an error (incorrect response: choice of wrong chest) or failed to respond (omission) within a fixed time limit (45s^20^) that trial was stopped and the horse was given another trial after being led back to the starting line. The number of trials required to learn to go towards an illuminated chest (correct response) to gain access to food was considered a measure of learning ability. Then, tests were conducted on the day following the training protocol in the same condition. Two test sessions of ten trials each were performed on two successive days (Test 1 and Test 2). For each session, the light stimulus (0.5 sec) was presented on each chest the same number of times (*i.e*. twice), but in a randomized order (*e.g*. 2-3-5-4-3-1-4-2-5-1, or 5-4-4-1-3-2-5-3-1-2, etc.). The attentional capacities of the subject were assessed by the “accuracy” (*i.e*. correct responses/total responses) of choosing the correct (*i.e*. illuminated) chest.

### Attention in a training context: lunge training task

Each mare was trained to obey an experimenter’s vocal commands during a lunge working task. Training included 10 daily 5-minute working sessions. Sessions occurred five days a week for two consecutive weeks. Before each session, all mares were equipped with a cavesson and a lunge rope (8 m long) and led from their home stalls to a familiar circular sand arena (diameter: 19.5 m). All mares were trained by the same experimenter (CR) giving vocal orders. The aim was to train the horses to obey the experimenter’s vocal commands such as ‘walk!’ (*i.e*. start to walk and continue to walk round the arena), or ‘trot!’ (*i.e*. start to trot and continue to trot round the arena). The observations were focused on mean latencies to respond to the different vocal cues and on failure to attend to signals as an indicator of inattention (*i.e*. sudden unrequested gait changes as ascending gait changes, *e.g*. canter instead of requested to trot)^37^. These behaviours were recorded using *ad libitum* sampling^29^.

Relationship between attention measurement in the training task and DT data were explored.

### Statistical analyses

Normality and homogeneity of variances were assessed by inspection of residuals and Shapiro–Wilk W tests^38^. As our data were not normally distributed, we applied non-parametric statistical tests^39^. We computed the coefficient of inter-individual variation (CV = standard deviation/mean × 100) for each attention variable^23^ to evaluate inter-individual variability.

Wilcoxon signed-rank two-tailed tests evaluated differences in attention between whale and conspecific stimuli during the DT. Numbers of horses reacting were compared between days and stimuli using Chi-square tests and variations of reaction times (including maximum reaction time scores) and durations of attention in relation to test day were evaluated using Friedman tests. The impact of test day was estimated by multiple pairwise comparisons using Wilcoxon signed-rank t-tests with FDR correction (‘false discovery rate’, adjusted alpha level, to avoid an inflated Type I error rate)^40^. Spearman’s correlation tests evaluated relationships between tests (*i.e*. DT, VAT; 5-CSRTT; Training task) and with subjects’ age. The impact of age was evaluated using Mann-Whitney U-tests to compare the older (>10 y.o) and younger (<10 y.o) mares. These analyses were computed with Statistica 7.1 software © (accepted *p* level at 0.05). Descriptive statistics are median and range (minimum-maximum).
